# Effects of intracerebroventricular anandamide administration on feed intake and milk yield of dairy cows

**DOI:** 10.3168/jdsc.2021-0185

**Published:** 2022-02-10

**Authors:** Björn Kuhla, Isabel van Ackern

**Affiliations:** Research Institute for Farm Animal Biology (FBN), Institute of Nutritional Physiology ‘Oskar Kellner', Wilhelm-Stahl-Allee 2, 18196 Dummerstorf, Germany

## Abstract

Among the endocannabinoids, *N*-arachidonylethanolamide (AEA; anandamide) plays a key role in regulating energy homeostasis and energy intake. Recent studies suggest the existence of a peripheral mechanism by which AEA increases feed intake in the short term and modulates whole-body energy metabolism in dairy cows. Here, we aimed to test the hypothesis that AEA has a long-lasting central effect in increasing feed intake that leads to an increase in milk yield of dairy cows. In the present pilot study, 3 nonpregnant Holstein dairy cows were equipped with an intracerebroventricular (i.c.v.) catheter. Cows were deprived from feed for 2 h and received either no injection or an i.c.v. injection of either 12 μg of AEA or DMSO (control), followed by measurement of feed intake for 10 h and milk yield. Administration of AEA increased 10-h dry matter intake (DMI) by between 1.13 and 2.06 kg, whereas 22-h DMI was only marginally altered. However, compared with the control treatment, AEA reduced daily milk yield by 0.3 to 1.4 L/d in all 3 cows. The results demonstrate that i.c.v. administration of 12 µg of AEA increased 10-h DMI but decreased daily milk yield by a central mechanism.

## Highlights

• Intracerebroventricular *N*-arachidonylethanolamide (AEA) injection increases short-term feed intake of cows.
• Intracerebroventricular injection of AEA has no long-term effect on feed intake.
• Intracerebroventricular AEA injection reduces daily milk production.

## Graphical Abstract

**Figure:**
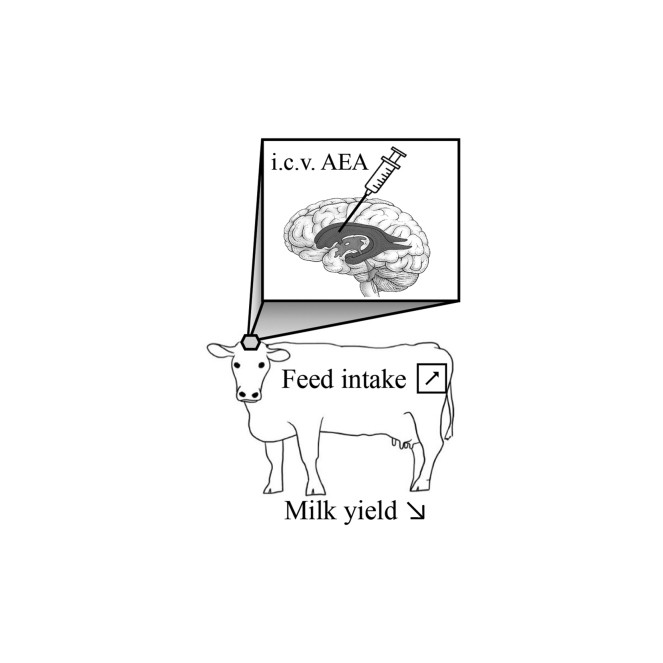
**Summary:** Improving feed intake in dairy cows could lead to health and economic benefits. Previous research has shown the existence of a peripheral mechanism for the regulation of feed intake by endocannabinoids, but recent work indicates that central mechanisms may also exist. Thus, we investigated the effect of an intracerebroventricularly injected endocannabinoid, *N*-arachidonylethanolamide (AEA), on feed intake and lactation performance of dairy cows. Cows temporarily increased feed intake but not daily milk production, suggesting that the surplus of energy was otherwise metabolically converted. Based on our understanding of this effect, we cannot recommend the use of endocannabinoids to maximize feed intake.

The endocannabinoid anandamide, also called *N*-arachidonylethanolamide (**AEA**), is a fatty acid neurotransmitter, which is synthesized in many tissues, including the brain. The dominant endocannabinoid receptor in the central nervous system is cannabinoid receptor 1 (**CB1**), whose activation plays a key role in regulating energy homeostasis and feed intake. Prior research has shown that CB1 receptors are expressed in many brain areas associated with the control of appetite, hunger, eating behavior, and energy metabolism, including the limbic system, brainstem, and hypothalamus of rats (Svízenská et al., 2008). Microinjection of 50 ng of AEA into the hypothalamic ventromedial nucleus (Jamshidi and Taylor, 2001), the administration of 35 and 140 ng (but not 25 ng) of AEA into the hypothalamic paraventricular nucleus (Chapman et al., 2012), and administration of 1 µg of AEA into the nucleus accumbens (Soria-Gómez et al., 2007) of rats increased food intake from 1.5- to 6-fold within 2 to 4 h following injection. However, the insertion of the needle tip directly into brain tissue may destroy a substantial number of cells located in these brain areas and thus their function. As an alternative administration route, intracerebroventricular (**i.c.v.**) injections into one of the two lateral ventricles or the third brain ventricle may prevent disruption of brain areas controlling feed intake. In this way, the chemical compound is administered into the cerebrospinal fluid (**CSF**) reservoir, from where it may enter the surrounding brain areas, including the hypothalamic and limbic nuclei. However, i.c.v. injections of 0.1, 1, and 10 µg of AEA did not increase food intake of rats within 4 h after administration (Gómez et al., 2002). The reason for the lack of effect on food intake after i.c.v. administration is not quite clear. It is possible that in different areas of the brain AEA exerts different effects on food intake, that the molality of the saline used as vehicle was too high, or that high doses of AEA suppress food intake. The latter assumption is supported by the finding that intrahypothalamic administration of 150 ng compared with 50 ng of AEA did not increase food intake (Jamshidi and Taylor, 2001), and that i.c.v. administration of 10 µg relative to 1 and 0.1 µg of AEA decreased food intake of rats (Gómez et al., 2002). Bimodal effects of AEA concentrations on food intake have also been reported in rainbow trout, with the higher doses of AEA (5 vs. 2 ng/100 g of BW) not affecting food intake levels (Díaz-Rúa et al., 2021).

In ruminants, there is little information about the involvement of AEA in feed intake regulation. The CB1 receptor was found to be expressed in various areas of the hypothalamus in dairy cows (Kuhla et al., 2020), and plasma AEA concentrations increased with the increase in feed intake during early lactation, suggesting that at least circulatory AEA is involved in the control of feed intake in ruminants. In support of the latter assumption, intraperitoneal (i.p.) administration of 5 µg AEA per kg of BW has been shown to increase short-term feed intake of dairy cows for 1 h following injection (van Ackern et al., 2021a). It has not yet been determined whether AEA has a central effect in increasing feed intake in dairy cows, whether this effect lasts longer than 1 h and, if so, whether it increases milk yield. Therefore, the aim of this study was to explore the effect of i.c.v. administration of AEA on feed intake of lactating dairy cows.

All experimental procedures were performed at the Research Institute for Farm Animal Biology (FBN), Dummerstorf, Germany, and were conducted in accordance with the ARRIVE guidelines (https://arriveguidelines.org) and the German Animal Welfare Act. The experiments were approved by the ethics committee of the State Government in Mecklenburg-Western Pomerania, Germany (Registration No. LALLF M-V 7221.3–1.1–010/17).

Three nonpregnant German Holstein dairy cows were randomly chosen from the FBN herd and adapted to tiestalls on a 2.43 × 1.56 m stand with rubber mats and a feed bin at 15°C for 3 d. Cows were >100 DIM and were fed a TMR twice daily that was composed of corn silage, grass silage, hay, barley straw, concentrate, mineral mix and lime, as described previously (Kennedy et al., 2021). The ration contained 10.5 MJ of ME/kg of DM and was formulated to meet the requirements according to the recommendations by the German Society for Nutrition Physiology (GfE, 2001). On d 3, animals were deprived of feed starting at 1500 h. After an overnight fast, BW was measured and metabolic BW calculated as BW^0.75^. Then, the cow's forehead was shaved and a catheter (Cavafix Certo m. Splittocan, 32 cm; B. Braun Melsungen AG) inserted into the jugular vein. Anesthesia was initiated with xylazine (0.2 mg/kg; Riemser Arzneimittel AG) and ketamine (2 mg/kg; Serumwerk Bernburg AG) and maintained by continuous i.v. infusion of 700 mg of xylazine and 7 g of ketamine per 500 mL of 5% glucose solution (Glucose 5% B. Braun Ecoflac Plus; B. Braun Melsungen AG). Cows were transferred to a surgery room and placed on the right site for the implantation of a cannula guide directed to the lateral brain ventricle, as described previously (Kuhla et al., 2010). Briefly, the shaved skin was disinfected with iodine and 70% ethanol before local analgesia was set via infiltration anesthesia (300 mg of procaine hydrochloride, 0.38 mg of epinephrine; Isocain ad us. vet; Selectavet Dr. Otto Fischer GmbH). A triangular incision (~5 × 5 cm) was made to expose the frontal bone. With the help of a stereotaxic apparatus, a 3.5-mm (diameter) hole was drilled in the frontal bone. A homemade stainless steel cannula guide was inserted, secured with screws on the frontal bone, and sealed with a septum. A needle with fitted stylet (120 mm long, 1.2 mm in diameter) was pushed through the septum to aspirate CSF after removal of the stylet. Successful CSF aspiration confirmed the correct placement of the cannula guide and allowed us to determine the depth to which the needle should be inserted into the lateral brain ventricle. Animals received analgesia (Melovem: 0.5 mg meloxicam/kg; Dopharma B.V.) and antibiotics (Trimethosel; sulfadimidine sodium, 14.4 mg/kg, and trimethoprim, 2.7 mg/kg; aniMedica GmbH) for 3 d. After recovery from surgery, animals were transported back to tiestalls and fed for ad libitum intake.

The first i.c.v. injection was performed 7 d after surgery. Feed residuals were removed from the feeding bin at 0700 h. Between 0700 and 0900 h, cows had no access to feed. To determine the effective AEA concentration, one cow was injected i.c.v. daily at 0900 h with 0, 1.5, 12, or 170 µg of AEA. For this, an adequate volume of an AEA solution (5 mg/mL, dissolved in ethanol; Tocris Bioscience) was taken and diluted with dimethyl sulfoxide (**DMSO**). Immediately after i.c.v. injection of 100 µL, the cow was given access to feed for ad libitum intake. Feed was prepared in one batch to reduce between-day variation, vacuum-packed in 40-kg plastic bags, and stored at 4°C before feeding. From 0900 to 1900 h, feed intake was measured as feed disappearance from the bin by an electronic registration device (PAARI) every 15 min. The 22-h feed intake was measured as a difference of feed mass provided at 0900 h and feed residues collected at 0700 h the next day.

Because the 12-µg dose of AEA was found to increase feed intake in the first cow studied, the following 3-d experimental injection protocol was applied to all cows. Each cow received i.c.v. control (**CON**; 100 µL of DMSO), 12 µg of AEA, or no injection on 3 d, in randomized order. Cows were randomly assigned to the treatment sequence. Individual feed intake was recorded as described above. Rectal temperature was measured in 24-h intervals using a thermometer with 10-cm insertion depth. A feed sample from each batch was taken to determine the DM content after drying at for 24 h at 60°C. Dry matter intake was calculated from feed intake and feed DM content. Cows were milked at 0630 and 1700 h to determine milk yield. Daily milk yield was calculated as the sum of the evening and subsequent morning milking.

Cumulative feed intake data was analyzed using the PROC MIXED model in SAS software (version 9.4, SAS Institute Inc.). The fixed effects were treatment (no, CON, AEA), time, and their interaction. The repeated statement included treatment and time, the type was unstructured, and subject was cow. Data for DMI and milk yield were compared between CON and AEA, and between no treatment and AEA using the paired *t*-test in JMP software (version 13, SAS Institute Inc.).

Administrations of 1.5 and 170 µg of AEA relative to 0 µg of AEA did not affect feed intake in the cow examined (data not shown). However, i.c.v. injection of 12 µg of AEA increased cumulative feed intake (Figure 1) and DMI (Table 1) during the 10-h postinjection period in all 3 cows (*P* < 0.05). The magnitude of DMI increase relative to CON injection 1 h postinjection was 1.13, 1.91, and 2.06 kg, respectively, which corresponds to a factor ranging between 1.1 and 1.3 (Table 1). Relative to CON injection, AEA administration reduced daily milk yield by 0.7 to 1.4 L/d (*P* < 0.05). However, at 22 h postinjection, DMI was not markedly different between AEA and CON treatments. The efficiency measure milk yield/10-h DMI ratio was 0.23 to 0.45 L/kg lower in AEA than in CON cows (*P* < 0.05), but milk yield/22-h DMI was not affected by AEA administration. Rectal temperatures measured before and 24 h after i.c.v. injection were not affected by treatment (mean ± SD; 38.2 ± 0.1).

**Figure 1:**
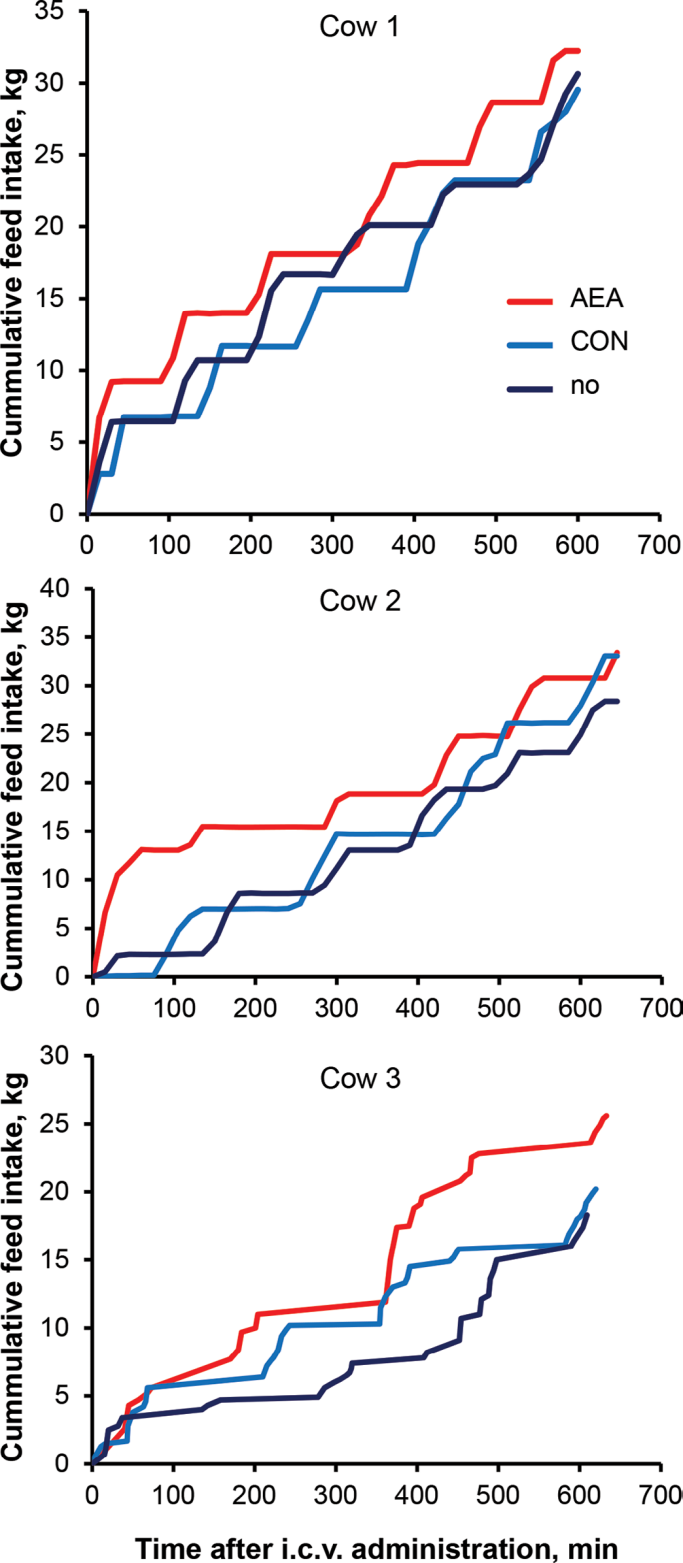
Cumulative feed intake measured every 15 min over a period of 10 h in 3 cows receiving no treatment or intracerebroventricular (i.c.v.) administration of dimethyl sulfoxide (sham injection; CON) or 12 µg of *N*-arachidonylethanolamide (AEA). Statistical analysis of the data from all 3 cows revealed an effect over time (*P* < 0.001), treatment (*P* < 0.05), and the interaction (*P* < 0.01).

**Table 1:** Body weight, DMI, and milk yield for 3 cows receiving no treatment or intracerebroventricular administration of CON (sham injection; dimethyl sulfoxide) or 12 μg of *N*-arachidonylethanolamide (AEA)

Cow	Treatment	BW, kg	DMI/10 h, kg	Milk yield, kg/d	Milk yield/DMI, (kg Ã— 10 h)/kg	DMI/22 h, kg	Milk yield/DMI, (kg Ã— 22 h)/kg
1	None	756.5	12.74	18.0	1.41	19.08	0.943
	AEA		13.41	17.2	1.28	19.08	0.901
	CON		12.28	18.6	1.51	19.17	0.971
2	None	628.5	10.37	23.2	2.24	18.03	1.286
	AEA		12.80	24.0	1.88	18.82	1.275
	CON		11.61	24.9	2.14	19.53	1.275
3	None	813.0	6.64	11.6	1.75	11.17	1.038
	AEA		9.00	10.7	1.19	10.98	0.974
	CON		6.94	11.4	1.64	10.83	1.053
	*P*-value						
	None—AEA		0.087	0.641	0.106	0.574	0.127
	CON—AEA		0.040	0.041	0.045	0.487	0.185

The aim of this study was to investigate whether i.c.v. injection of AEA increases feed intake of lactating dairy cows. We found that i.c.v. administration of 12 µg of AEA stimulates feed intake, at least during the first 10 h after injection in all 3 cows. The BW of the cows ranged between 628 and 813 kg, which implies an effective dosage of 15 to 20 pg of AEA/g of BW. In rats, the effective dosage was found to be approximately 10- to 20-fold higher when administered into the hypothalamus; for example, 150 to 200 pg/g of BW (Jamshidi and Taylor, 2001) and 420 pg/g of BW (Chapman et al., 2012). The major reason for this apparent discrepancy regarding effective dosage is likely the lower brain weight:BW ratio, which is about 1:1,000 for Holstein cows (Ballarin et al., 2016) and 1:100 in 60-d-old male Wistar rats (Song et al., 2018). As a result, the biologically effective dose eliciting an increase in feed intake in rats and cows amounts to about 25 ng of AEA/g of brain weight, regardless of whether AEA is administered into the hypothalamus or intracerebroventricularly. However, the 170-µg i.c.v. AEA injection (i.e., the 14-fold-higher dose) did not increase feed intake in the one cow studied. This result is in line with earlier findings by Díaz-Rúa et al. (2021) and Jamshidi and Taylor (2001), showing that high AEA doses do not elicit changes in food intake, whereas low doses induce orexigenic effects in rainbow trout and rats.

Recently, we showed that i.p. injection of 5 µg of AEA per kg of BW increased feed intake in dairy cows only within the first hour postinjection, and not during subsequent hourly intervals (van Ackern et al., 2021a). This short-term-only response was explained by the short half-life of AEA, although increased plasma AEA concentrations were still detected 2.5 h after i.p. injection (van Ackern et al., 2021b). The short-lasting but immediate AEA effect on feed intake increase could also be seen under conditions of i.c.v. administration in the current study, at least in cows 1 and 2, whereas cow 3 gained higher cumulative feed intake only after 1.5 h with AEA compared with CON administration. It is possible that the metabolic rate in cow 3 was not as high as that in the other 2 cows, as indicated by the level of milk production, which was approximately half that of cows 1 and 2. Reduced metabolic turnover may have enhanced the half-life of AEA in cow 3; however, to our knowledge, no study has demonstrated to what extent the metabolic rate influences the half-life of AEA. Further reasons for the delayed increase in feed intake in cow 3 compared with cows 1 and 3 may be different ceramide and protease levels, both known to be affected by surgery and to increase the half-life of AEA (Mallet and Beninger, 1998; Di Scala et al., 2017). However, an increased half-life of AEA may also explain the higher percentage increase in DMI of cow 3 within the 10-h monitoring period relative to no treatment or CON administration.

The higher DMI during the first hour after i.p. AEA administration did not affect 24-h DMI or milk yield (van Ackern et al., 2021a). Herein, we report that i.c.v. AEA administration also did not influence 22-h DMI, which can be explained by the limited half-life of AEA. In contrast to i.p. AEA administration, i.c.v. AEA administration reduced milk yield, as well as the milk yield/22-h DMI ratio and the milk yield/10-h DMI ratio, which indicates that the surplus of the dietary energy intake during the first 10 h was not used to increase milk synthesis. Various factors may explain this effect. An increase in DMI reduces feed digestibility and thus digestible energy intake, which may limit a parallel increase in metabolizable energy intake. More likely, however, is that the surplus of energy ingested was oxidized and spent as heat, because it has been demonstrated that intrahypothalamic AEA injection increased the respiratory quotient of rats (Chapman et al., 2012), and that i.p. AEA administration increased dietary carbohydrate oxidation and metabolic heat production in dairy cows (van Ackern et al., 2021a). The extent of carbohydrate utilization could not be measured in the present study, but its increase may explain the decline in milk yield after AEA administration. In addition to stimulating carbohydrate catabolism, endocannabinoids may exert hormone-like effects supporting anabolic processes such as increased de novo fatty acid synthesis in liver (Purohit et al., 2010) or adipose tissue lipogenesis (Myers et al., 2021). In dairy cows, AEA reduced whole-body fat oxidation (van Ackern et al., 2021a). Thus, we cannot exclude the possibility that a greater portion of dietary energy intake after i.c.v. AEA administration was directed to accrete body mass instead of being used for milk synthesis, although the accretion of body mass is hard to measure on an hourly basis.

This pilot study demonstrated that i.c.v. administration of 12 µg of AEA increased feed intake for 10 h but did not affect feed intake after 22 h, although it reduced daily milk yield. The milk yield:DMI ratios were lower for AEA than for CON or no treatment, demonstrating that the surplus of energy was not used for milk synthesis but rather otherwise metabolically converted. In future experiments, repeated injections or continuous infusion of AEA should be considered to account for its presumed rapid degradation and to study the long-term effects of AEA on metabolic traits of cows.
